# A potentially lifesaving error: unintentional high-dose adrenaline administration in anaphylaxis-induced cardiac arrest; a case report

**DOI:** 10.1186/s12245-024-00663-9

**Published:** 2024-06-28

**Authors:** Felix Patricius Hans, Leo Benning, Jan-Steffen Pooth, Hans-Jörg Busch

**Affiliations:** University Emergency Department, Medical Center – University of Freiburg, Faculty of Medicine, University of Freiburg, Freiburg, Germany

**Keywords:** Cardiopulmonary resuscitation, Advanced cardiac life support, Anaphylaxis, Neoplasms, Medical errors, Emergency medicine, Crew resource management, Healthcare

## Abstract

**Background:**

Cardiopulmonary resuscitation is a crucial skill for emergency medical services. As high-risk-low-frequency events pose an immense mental load to providers, concepts of crew resource management, non-technical skills and the science of human errors are intended to prepare healthcare providers for high-pressure situations. However, medical errors occur, and organizations and institutions face the challenge of providing a blame-free error culture to achieve continuous improvement by avoiding similar errors in the future. In this case, we report a critical medical error during an anaphylaxis-associated cardiac arrest, its handling and the unexpected yet favourable outcome for the patient.

**Case presentation:**

During an out-of-hospital cardiac arrest due to chemotherapy-induced anaphylaxis, a patient received a 10-fold dose of epinephrine due to shortcomings in communication and standardization via a central venous port catheter. The patient converted from a non-shockable rhythm into a pulseless ventricular tachycardia and subsequently into ventricular fibrillation. The patient was cardioverted and defibrillated and had a return of spontaneous circulation with profound hypotension only 6 min after the administration of 10 mg epinephrine. The patient survived without any residues or neurological impairment.

**Conclusions:**

This case demonstrates the potential deleterious effects of shortcomings in communication and deviation from standard protocols, especially in emergencies. Here, precise instructions, closed-loop communication and unambiguous labelling of syringes would probably have avoided the epinephrine overdose central to this case. Interestingly, this serious error may have saved the patient’s life, as it led to the development of a shockable rhythm. Furthermore, as the patient was still in profound hypotension after administering 10 mg of epinephrine, this high dose might have counteracted the severe vasoplegic state in anaphylaxis-associated cardiac arrest. Lastly, as the patient was receiving care for advanced malignancy, the likelihood of termination of resuscitation in the initial non-shockable cardiac arrest was significant and possibly averted by the medication error.

## Background

Sudden cardiac death is among the top three leading causes of death in developed countries. Emergency medical service (EMS) providers treat between 28 and 244 cardiac arrests per 100,000 inhabitants per year as out-of-hospital cardiac arrests (OHCA) worldwide [1]. In-hospital cardiac arrest (IHCA), at the same time, is a relevant driver of mortality in admitted patients, with an incidence of 1.5 patients per 1,000 [2]. Anaphylaxis-related cardiac arrests are highly infrequent events that occur in and out of hospitals (0,04/100,000 or 0.12% of OHCA) [3, 4]. While anaphylaxis-associated OHCA is triggered mostly by insect stings and food [5], anaphylaxis-associated IHCA is mainly caused by contrast and chemotherapy agents [4].

Cardiopulmonary resuscitation (CPR) and advanced life support (ALS) are core skills on which acute care providers receive training [6]. Although highly standardized, CPR situations pose a challenge, especially to ad-hoc teams in hospitals or the field. Epinephrine is among the crucial medications in both anaphylaxis and cardiac arrest. However, the dosage and route of application significantly differ depending on the clinical state. As a result, barriers to administering adequate doses of epinephrine frequently potentially result in deleterious under- and overdosing of epinephrine [7–10]. In states of anaphylactic shock, driven by fluid extravasation and vasodilation, the second-line therapy after epinephrine is the administration of crystalloid fluids (0.5–5 l) [11].

Crew resource management (CRM) and the analysis of human factors deal with the nature of human perception in high-pressure environments, the laws of communication and with the science of medical errors [12, 13]. Therefore, CRM elements are crucial in clinical and preclinical algorithms to ensure the best possible quality of care, especially in high-risk-low-frequency events like cardiac arrests [14]. Often, an error occurs from the convergence of multiple contributing factors and may harm patients (immediate effect of wrong or missed action) as well as health care providers (disciplinary action, blame, loss of trust, job leave) [15]. Environments with underdeveloped institutional handling of errors may lead to underreporting of mishaps, near-misses and errors, fostering an even more harmful environment for patients [16]. Improving communication and medication safety, therefore, is among the ten goals of the Joint Commission for Patient Safety [15, 17, 18].

Here, we report the case of an anaphylaxis-associated OHCA in an oncologic private practice, where a severe medication error occurred due to shortcomings in communication and standardization. Despite this error, the patient had a favourable outcome and could consent to the case presentation. The report is drafted according to the CARE reporting guideline [19].

## Case presentation

A 66-year-old woman was treated in an outpatient clinic/private practice for uterine serous carcinoma. On that day, the second dose of paclitaxel was scheduled to be administered via a venous port catheter system after an initial dose was administered 21 days before without any complications. A blood sample drawn before the start of the paclitaxel therapy showed no abnormalities except mild anemia (10.8 g/dl). On a routine basis, the patient received 20 mg of dexamethasone p.o., 150 mg of ranitidine p.o. and 2 mg of clemastine fumarate i.v. via the port catheter system. Subsequently, the nursing staff started a drip of 266 mg of paxlitaxel. The patient complained about shortness of breath (SOB) and nausea 10 min into the therapy. The oncologist was called to the patient and administered another 20 mg of dexamethasone i.v. and another push dose of 2 mg clemastine fumarate i.v. via the port catheter system. The patient was then transferred to a separate cubicle with a stretcher.

Upon arrival in the cubicle, the patient remained awake and still complained about SOB and nausea. The outpatient clinic’s team administered oxygen and called the emergency dispatch under the impression of an anaphylactic shock. In Germany, the EMS are dispatched in a rendezvous system with a doctor’s vehicle, which will assist in special circumstances by bringing a pre-hospital emergency physician to the site of an accident or illness [20, 21]. In this case, an emergency physician was dispatched together with an ambulance to the code “anaphylaxis”. By that time, the patient had a loss of peripheral pulses, lost consciousness and had a non-invasive blood pressure (NIBP) of 60/40mmHg. An epinephrine drip was prepared by the outpatient clinic (of which the dose could not be determined in hindsight). Before the administration of the drip, the patient went into cardiac arrest (t = 0 min), and CPR was started immediately, as the outpatient clinic’s staff had coincidentally performed basic life support training the day before.

On arrival of the EMS (t = 8 min), the patient presented with a Glasgow Coma Scale (GCS) of 3, all vital signs absent under CPR from the outpatient clinic staff. EMS took over CPR and Bag-Mask-Ventilation (BMV) and attached a heart monitor (t = 8 min) showing a pulseless electrical activity (PEA: Brady-asystole with broad QRS complexes at a rate of approximately 10/min). According to ALS Guidelines, the team intended to apply 1 mg of epinephrine as a non-shockable rhythm was present [2, 6]. As to the EMS it was unclear if the infusion connected to the patient’s port catheter system contained the potentially triggering substance, the drip was removed and replaced by 500 ml of a balanced crystalloid solution (t = 9 min). A 10 ml syringe was handed to the emergency physician, stating “Epinephrine is ready!”, and the epinephrine was administered via the port system (t = 10,5 min). The emergency physician returned the syringe to the paramedic, asking for “another milligram of epinephrine” to be administered within 3–5 min (according to ALS guidelines). The paramedic responded that he had just handed 10 mg in 10 ml to the emergency physician, and it became clear to the team that 10 mg of epinephrine had been applied directly to the port catheter. Almost immediately, the patient developed pulseless ventricular tachycardia (pVT). She received defibrillation with 200 J (biphasic, t = 12 min), and after 2 min of CPR, the patient was in ventricular fibrillation (vFib). After defibrillation with 200 J (biphasic, t = 14 min), the team performed a rhythm and pulse check after another 2 min of CPR and detected sinus tachycardia (136/min) and a central pulse (t = 16 min).

The return of spontaneous circulation (ROSC) was announced, and an evaluation according to the ABCDE mnemonic took place approximately 6 min after applying 10 mg of epinephrine. The patient presented with an open airway, ongoing BMV and a spontaneous respiratory rate of 14/min. End-tidal CO2 (etCO2) showed values of around 35mmHg, and NIBP showed profound hypotension (73/45 mmHg). Subsequent actions included the repetitive administration of norepinephrine push doses of 10 µg and endotracheal intubation (due to persistent GCS = 3; after administering 10 mg midazolam, 10 mg morphine and 20 mg etomidate).

Transport to the nearby cardiac arrest centre (CAC [22]) was uneventful. The patient was handed over to the cardiac arrest receiving team at t = 53 min. Norepinephrine (13 mcg/min) was administered with another 2000 ml of crystalloid fluids during the first 120 min after OHCA to counteract the persisting state of shock. A multi-region computer tomography (CT) revealed fractures of the costae 3–5 on the right and 3–6 on the left side with associated tension pneumothorax on the left side, which was decompressed with a chest tube in the emergency department. Cranial CT showed no abnormalities. Given the precise line of events, a normal ECG and only slightly elevated troponin T levels (67.1 ng/l) upon arrival, no coronary angiography was performed. According to local post-resuscitation care, outcome prediction after OHCA relies on cerebral imaging, CPR details (i.e. no-flow-time, underlying rhythm, etc.) and the biomarkers Neuron-Specific Enolase (NSE) and Serum S-100 β protein (S100), that were measured on arrival [23, 24]. The NSE level was 47.6 µg/l, the S100 level was 5.730 µg/l, and the patient received cooling to 33 °C for 24 h. The maximum C - reactive protein was measured with 167.5 mg/ on day five after CPR, while aspiration pneumonia was treated with antibiotics (ampicillin/sulbactam).

Extubation took place after three days. The patient reported having nightmares for three days and hallucinations of black figures standing beside the bed. All symptoms eased on day four after extubation. The chest tube was removed on day five after CPR, and the patient was transferred to a primary hospital (closer to the patient’s home) after nine days in the intensive care unit (ICU). The primary hospital discharged the patient home after another six days of inpatient care without any residuals (Cerebral Performance Category (CPC) 1) except for newly diagnosed hypertension.

## Discussion and conclusions

In the above case, an excessively high dose of epinephrine was directly administered through central venous access in a non-shockable OHCA that was considered anaphylaxis-associated and healthcare-related [2]. It is known that the incidence of anaphylaxis-associated cardiac arrest is very low [2–5] and that epinephrine may lead to higher rates of ROSC but does not foster beneficial neurological outcomes [25]. In this case, the conversion to a shockable rhythm, potentially induced by high-dose epinephrine, led to an immediate change of the ALS management, as the patient could be defibrillated as a result and had ROSC. Although it is widely accepted that shockable rhythms in cardiac arrest show higher survival rates and better CPC scores [26, 27], no causation can be postulated, even if the administration of epinephrine was followed by immediate conversion of rhythm in this case. Nevertheless,, given the physiologic half-life of epinephrine between 3 and 20 min [28, 29], the BP measured 6 min after the administration of 10 mg epinephrine via central venous access was surprisingly low with only 73/45 mmHg, indicating severe, persistent vasoplegic shock. The necessity of push doses of norepinephrine and administering 2.5 l of crystalloid fluids in the first 120 min after OHCA underscore this persistent state of shock. Yet, the discovered tension pneumothorax might have led to the development of an additional obstructive shock (due to chest compressions or ventilation) and, therefore, might have contributed to the persisting shock. In hindsight however, the high dose of epinephrine might have counteracted the shock states without causing relevant side effects.

Given the patient’s medical history with the respective clinical gestalt (advanced cancer state, therapy-associated BMI of 18 and hair loss), we assume that adherence to the guidelines by administering 1 mg of epinephrine might not have led to a ROSC after only 6 min. The EMS team would likely have considered an early termination of resuscitation (TOR) under those alternate circumstances.

In a structured debriefing following the CRM concept, the medical error was narrowed down to a divergence of standard dosages between the hospital environment and the local EMS. By standard, the physician in charge used epinephrine 1:10,000 (0.1 mg/ml), whereas the EMS standard concentration is 1:1,000 (1 mg/ml). Clearly, the emergency physician should have been aware of this fact and asked for a labelled syringe with a clear statement of the intended dose. This shortcoming led to the administration of a potentially harmful injection of an epinephrine overdose, which presumably saved the patient’s life in this case.

Given the descriptive nature of this work as a case report, the clear limitation is the lack of generalizability, and the finding does not imply a routine deviation from guidelines. Especially the previously described absence of clinical benefits from high doses of adrenalin in cardiac arrest [30, 31] and its potentially harmful effects in anaphylaxis (i.e. myocardial infarction, pulmonary edema, death) [9] should lead to critically interpretating the events described here.

As the incidence of anaphylaxis-associated OHCA is very low [3–5] our case might nonetheless describe important findings on the topic. Firstly, the management of anaphylaxis is often characterized by inappropriate dosing and timing of epinephrine as a first-line medication [4, 9, 10]. This might indicate a need for high-fidelity simulation and training for anaphylaxis and CPR in special circumstances [2, 32] and for organizational measures like standardization of code medications and prefilled or pre-labelled syringes [8, 33]. Second, crew resource management elements (e.g. closed-loop communication, read-backs, call-outs) [32, 33] should be a bedrock component in professional development for all healthcare workers. Additionally, these measures must be embedded in error-preventing environments on organizational levels [16, 34]. As over two-thirds of anaphylaxis-associated IHCA occurred in malignancy patients [4], further research should systematically analyze the mechanisms and possible adaptations of guidelines in anaphylaxis-associated cardiac arrest to avoid premature TOR in those patients.

## Data Availability

No datasets were generated or analysed during the current study.

## Abbreviations

BMV Bag: Mask Ventilation
C: Celsius
CPR: Cardiopulmonary Resuscitation
ROSC: Return of Spontaneous Circulation
IHCA: In-Hospital Cardiac Arrest
i.v.: Intravenous
J: Joule
l: Litre
mcg: Microgram
mg: Milligram
min: Minute
ml: Millilitre
OHCA: Out-Of-Hospital Cardiac Arrest
p.o.: By Mouth
t: Time
TOR: Termination of Resuscitation
